# Sensitivity of Osteosarcoma Cells to Concentration-Dependent Bioactivities of Lipid Peroxidation Product 4-Hydroxynonenal Depend on Their Level of Differentiation

**DOI:** 10.3390/cells10020269

**Published:** 2021-01-29

**Authors:** Suzana Borovic Sunjic, Ana Cipak Gasparovic, Morana Jaganjac, Gerald Rechberger, Andreas Meinitzer, Tilman Grune, Sepp D. Kohlwein, Branka Mihaljevic, Neven Zarkovic

**Affiliations:** 1 Laboratory for Oxidative Stress (LabOS), Division of Molecular Medicine, Rudjer Boskovic Institute, HR-10000 Zagreb, Croatia; Suzana.Borovic.Sunjic@irb.hr (S.B.S.); Ana.Cipak.Gasparovic@irb.hr (A.C.G.); Morana.Jaganjac@irb.hr (M.J.); Branka.Mihaljevic@irb.hr (B.M.); 2 Institute of Molecular Biosciences, Bio TechMed-Graz, University of Graz, 8010 Graz, Austria; gerald.rechberger@uni-graz.at (G.R.); seppie99@gmail.com (S.D.K.); 3 University Clinic of Traumatology, University of Graz, 8010 Graz, Austria; andreas.meinitzer@klinikum-graz.at; 4 Department of Molecular Toxicology, German Institute of Human Nutrition Potsdam-Rehbruecke, 14558 Nuthetal, Germany; scientific.director@dife.de; 5 Department of Physiological Chemistry, Faculty of Chemistry, University of Vienna, A-1090 Vienna, Austria

**Keywords:** 4-hydroxynonenal, human osteosarcoma, differentiation, proliferation, apoptosis, ALP, GSH, GST, proteasomal activity, HNE-protein adducts, fatty acid, C20:3

## Abstract

4-Hydroxynonenal (HNE) is a major aldehydic product of lipid peroxidation known to exert several biological effects. Normal and malignant cells of the same origin express different sensitivity to HNE. We used human osteosarcoma cells (HOS) in different stages of differentiation in vitro, showing differences in mitosis, DNA synthesis, and alkaline phosphatase (ALP) staining. Differentiated HOS cells showed decreased proliferation (^3^H-thymidine incorporation), decreased viability (thiazolyl blue tetrazolium bromide-MTT), and increased apoptosis and necrosis (nuclear morphology by staining with 4′,6-diamidino-2-phenylindole-DAPI). Differentiated HOS also had less expressed c-MYC, but the same amount of c-FOS (immunocytochemistry). When exposed to HNE, differentiated HOS produced more reactive oxygen species (ROS) in comparison with undifferentiated HOS. To clarify this, we measured HNE metabolism by an HPLC method, total glutathione (GSH), oxidized GSH (ox GSH), glutathione transferase activity (GST), proteasomal activity by enzymatic methods, HNE-protein adducts by genuine ELISA and fatty acid composition by GC-MS in these cell cultures. Differentiated HOS cells had less GSH, lower HNE metabolism, increased formation of HNE-protein adducts, and lower proteasomal activity, in comparison to undifferentiated counterpart cells, while GST and oxGSH were the same. Fatty acids analyzed by GC-MS showed that there is an increase in C20:3 in differentiated HOS while the amount of C20:4 remained the same. The results showed that the cellular machinery responsible for protection against toxicity of HNE was less efficient in differentiated HOS cells. Moreover, differentiated HOS cells contained more C20:3 fatty acid, which might make them more sensitive to free radical-initiated oxidative chain reactions and more vulnerable to the effects of reactive aldehydes such as HNE. We propose that HNE might act as natural promotor of decay of malignant (osteosarcoma) cells in case of their differentiation associated with alteration of the lipid metabolism.

## 1. Introduction

Oxidative stress occurs in cells as a consequence of oxygen metabolism. The reactive oxygen species (ROS) produced during oxidative stress may damage intracellular components, including lipids causing chain reaction of lipid peroxidation. Reactive aldehydes, end-products of lipid peroxidation, are involved in the onset and progression of many diseases such as cardiovascular diseases, neurodegeneration, fibroproliferative disorders, cancer, etc. [1,2,3]. Unregulated or prolonged production of ROS as well as of reactive aldehydes may influence cancer development and progression not only directly as a mutagen but also through modification of gene expression [4]. Tumor cells are under persistent mild oxidative stress which seems to be beneficial for them, increasing their metastatic potential and genetic instability, thus helping tumor cells to survive and progress [5,6]. It is well documented that antioxidant defense systems are altered in tumorigenesis promoting tumor progression [7]. On the other hand, severe oxidative stress is harmful for tumor cells; additional ROS production caused by chemotherapy, irradiation, or innate immune response is cytotoxic and leads to cellular destruction [1,8]. Nowadays, numerous strategies in cancer therapy rely on inducing excessive ROS, promoting lipid peroxidation and ferroptosis [9]. Osteosarcoma cells are often resistant to oxidative stress induced by chemotherapy [10,11]. 

The focus of our research is differences in response of differentiated and undifferentiated cells to lipid peroxidation, in particular with respect to the role of second messenger of ROS 4-hydroxynonenal (HNE). HNE alters cellular functions such as membrane integrity, mitochondrial respiration, etc.; but it is also a signaling molecule modulating expression of stress genes [4,12].

Normal and malignant cells of the same origin differ in sensitivity to oxidative stress. We have previously described differential sensitivity to HNE of CEM-NKR leukemic cells and normal human peripheral mononuclear cells, where HNE inhibited the growth of malignant cells, but not normal [13]. The same result is observed when normal and malignant mesenchymal cells are analyzed. Normal human osteoblasts and WI38 fibroblasts are less sensitive to HNE than 143 B and HOS osteosarcoma cells [14]. In this article we wanted to clarify whether differentiation of the mesenchymal cells would influence sensitivity to HNE. HOS cell line is able to differentiate in cell culture, so it is used for this purpose [15]. We analyzed HOS cells with different degrees of differentiation in respect to their sensitivity to HNE, ability to detoxify HNE, GSH content, GST activity, and proteasomal activity. We also analyzed the composition of fatty acids in those cells as they serve as a substrate for oxidation, increasing the damage to the cells.

## 2. Materials and Methods

### 2.1. HOS Cell Line

The human osteosarcoma cell line HOS was obtained from American Type Culture Collection (ATCC). Cells were maintained in DMEM (Dulbecco’s modified eagle’s medium, Sigma-Aldrich, St. Louis, MO, USA) with 5% (*v*/*v*) fetal calf serum (FCS, Sigma-Aldrich, St. Louis, MO, USA) in T75 cell culture flasks (Sarstedt, Nümbrecht, Germany), in an incubator (Heraeus, Hanau, Germany) at 37°C, with a humid air atmosphere containing 5% CO_2_. For the experiments, cells were detached from semiconfluent cultures with a 0.25% (*w*/*v*) trypsin solution for 5 min. Viable cells (upon trypan blue exclusion assay) were counted on a Bürker-Türk hemocytometer and used for experiments. All cell culture experiments were performed in such conditions if not stated otherwise for each particular experiment. 

In order to differentiate the HOS cell culture, cells were grown for 10 days without detaching and medium was changed every second day. After this period, cells were used for experiments, and are referred as differentiated HOS. Likewise, HOS cells which were maintained in semiconfluent state are referred as undifferentiated HOS. 

### 2.2. Alkaline Phosphatase Staining

Undifferentiated and differentiated HOS were washed with phosphate buffered saline (PBS) pH 7, fixed with 4% buffered paraformaldehyde (Kemika, Zagreb, Croatia) for 1 h at 4 °C. Before staining cells were washed three times with PBS and three times with 0.9% NaCl. Cells were then incubated with 500 µL/cm^2^ of stain (naphthol phosphate AsMx (Sigma-Aldrich, St. Louis, MO, USA), 4.4 mg sodium borate (Kemika, Zagreb, Croatia), 0.9 mg Fast Blue RR (Sigma-Aldrich, St. Louis, MO, USA), 36 µg magnesium sulphate (Kemika, Zagreb, Croatia) in 1 mL of 0.9% NaCl (Kemika, Zagreb, Croatia) pH 8.5) at 37 °C for 30 min in incubator (Heraeus, Hanau, Germany). Cells of osteogenic origin have alkaline phosphatase (ALP) which increase with cell differentiation [15,16]. Yellow stain was metabolized to blue deposits if ALP was present in cells. Number of ALP-positive nodules was counted in cell cultures. 

### 2.3. ^3^H-Thymidine Incorporation Assay

The rate of radioactive ^3^H-thymidine incorporation into DNA was used to measure proliferative activity of differentiated and undifferentiated HOS. Differentiated and undifferentiated HOS were detached, seeded 2 × 10^4^ cells in 96-well microtiter plates (Greiner Bio-One GmbH, Frickenhausen, Germany) in a final volume of 200 µL and were cultured for 48 h. For testing effects of HNE on cell proliferation, HOS were treated with different concentrations of HNE (0, 1, 5, or 10 μM) for 48 h. After the first 24 h 0.1 µCi of radioactive thymidine ([6-^3^H] thymidine, 1 mCi/mL, Amersham Biosciences, Amersham, UK) was added to each well. The cells were harvested on glass filters in a cell harvester (Skatron, Lier, Norway) and ^3^H-thymidine incorporation was measured using a liquid scintillation β-counter (Beckman 7400, Brea, CA, USA) [15]. 

### 2.4. GSH Measurement

Differentiated and undifferentiated HOS were detached, washed with PBS and frozen immediately in liquid nitrogen. Total and oxidized glutathione were determined by method of Teitze [17]. Briefly, 1 × 10^6^ cells were resuspended in 50 µL of 0.1 M phosphate buffer with 5 mM EDTA, pH 7.5. Next, 5 µL of such sample was resuspended in 250 µL of phosphate buffer, vortexed, centrifuged for 7 min at 500× *g* and supernatant was taken for analysis. GSH standards were prepared from freshly prepared 1 mM GSH (Sigma-Aldrich, St. Louis, MO, USA) stock solution. A total of 10 µL of standards and samples were pipetted to 96-microwell plates with 50 µL phosphate buffer and background absorbance was measured at 415 nm (Easy-Reader 400 FW, SLT Lab Instruments GmbH, Salzburg, Austria). After that, 50 µL of 0.948 mg/mL DTNB (5,5’-dithio-bis-2-Nitrobenzoic Acid, Sigma-Aldrich, St. Louis, MO, USA), 50 µL of glutathione reductase, 8 U/mL and 0.667 mg/mL NADPH were added. The reaction mix was incubated for 3 min at room temperature, when absorbance was measured at 415 nm. The cellular GSH content was calculated from the standard curve. The same cell lysates were used for determination of oxidized GSH. The procedure was the same, only with 0.02 M NEM (N-ethylmaleimid, Sigma-Aldrich, St. Louis, MO, USA) in phosphate buffer used in the second step cells were resuspended. NEM blocks free GSH and leaves only oxidized GSH in cell sample [17]. The amount of GSH was calculated according to amount of cellular proteins determined by Bradford assay [18]. 

### 2.5. GST Activity

Differentiated and undifferentiated HOS were detached, washed with PBS, and frozen immediately in liquid nitrogen. GST was determined by enzymatic method [19]. Samples of 1 × 10^6^ cells were lysed with 500 μL of distilled water by vortexing for 2 min. Cell lysates were centrifuged at 500× *g* for 7 min and supernatant was used for analysis. In total, 25 μL of sample or GST (Sigma-Aldrich, St. Louis, MO, USA) standards were added into plastic cuvette followed by 750 μL of 100 mM KH_2_PO_4_ (Kemika, Zagreb, Croatia) pH 6.25 and incubated at room temperature for 5 min. Background absorbance was measured at 340 nm (Shimatzu, Kyoto, Japan). Then, 100 μL of 7.5 mM 1-choloro-2,4-dinitrobenzene (CDNB, Sigma-Aldrich, St. Louis, MO, USA) was added immediately followed by 100 μL of 10 mM GSH (Sigma-Aldrich, St. Louis, MO, USA). Samples were incubated at room temperature for 15 min and second absorbance was measured at 340 nm. First absorbance was taken from the second one and results were calculated from standard curve. The amount of GST activity was calculated according to amount of cellular proteins determined by Bradford assay [18]. 

### 2.6. Cell Viability Assay

Thiazolyl blue tetrazolium bromide (MTT) was used to measure mitochondrial activity which reflects viability of the cells. Differentiated and undifferentiated HOS were detached; the cells were plated at density of 2 × 10^4^/well in quadruplicates into 96-microwell plates (Greiner Bio-One GmbH, Frickenhausen, Germany) in final volume of 200 µL/well and incubated for 24 h in DMEM with 5% of FCS containing different concentrations of HNE (0, 1, 2.5, 5, 10, or 20 μM). After 24 h, the medium was removed and replaced with 200 μL of Hank’s balanced salt solution without phenol red and 20 μL of the MTT substrate solution (EZ4U, Biomedica, Vienna, Austria). Cells were incubated at 37 °C for 2 h and the absorbance was measured at 450 nm with 620 nm as a reference wavelength [20] on a plate reader (Easy-Reader 400 FW, SLT Lab Instruments GmbH, Worgl, Austria). 

### 2.7. Cell Treatments with HNE for Free HNE Analysis, GSH Analysis, and HNE-ELISA Analysis 

Undifferentiated and differentiated HOS were detached, cells were washed twice with sterile Krebs-Henseleit buffer, and suspension of 1 × 10^6^ cells/mL was pipetted into sterile glass tubes. Cells were then treated with HNE at final concentration of 20 µM (20 nmol/10^6^ cells) and incubated at 37 °C in incubator for 120 min unless specified differently. This particular HNE:cells ratio was chosen because it corresponds to 2 µM HNE in experiments in microwell plates we used through this work. 

For cell viability by Trypan blue exclusion assay, cells were centrifuged at 200× *g* for 5 min (Heraeus, Hanau, Germany), the supernatants were discarded, and cell viability was determined immediately by Trypan blue.

For free-HNE analysis, samples were taken at different time points (30, 60, 90, and 120 min), and centrifuged at 200× *g* for 5 min (Heraeus, Hanau, Germany). The supernatants were mixed with an equal volume of acetonitrile/acetic acid (24:1 *v*:*v*, (Merck, Darmstadt, Germany), centrifuged, and the supernatants were further stored at −80 °C for free HNE analysis on HPLC. 

For the HNE-ELISA (HNE-binding studies), cells were centrifuged at 200× *g* for 5 min (Heraeus, Hanau, Germany) the supernatants were discarded, and the cell pellets were washed twice with PBS, centrifuged, and stored at −80 °C till analyses. 

For the GSH analysis, cells were centrifuged at 200× *g* for 5 min (Heraeus, Hanau, Germany), the supernatants were discarded, and the cell pellets were washed twice with PBS, centrifuged, and stored at −80 °C till the analyses. 

### 2.8. Determination of Free HNE by HPLC Method

HNE standards were prepared by serial dilution from 1M HNE stock solution stored at −20 °C. Samples stored at −80 °C where thawed prior to analysis. After thawing, samples were vortexed, centrifuged at 500× *g* 20 min at 4 °C (Sigma Laborzentrifugen GmbH, Osterode am Harz, Germany) and analyzed by HPLC as already described [21]. The samples (20 μL) were injected into the HPLC system (Beckman System Gold Solvent module 128 with the UV Detector, Beckman, Brea, CA, USA and a Midas Spark Holland autosampler, Spark Holland, Emmen, The Netherlands). The mobile phase consisted of acetonitrile/water (42:58, *v*/*v*) (Merck, Darmstadt, Germany). The flow was set to 0.9 mL/min and the absorbance at 223 nm. The samples were analyzed on a Beckman Ultrasphere ODS, 5 μm, 4.6 × 150 mm column (Beckman Coulter, Brea, CA, USA) at room temperature. 

### 2.9. Determination of HNE-Protein Adducts by HNE-ELISA

Cell pellets were lysed with 400 μL of lysis buffer (6M guanidine, 0.6055 g TRIS, 0.8766 g NaCl in 100 mL H_2_O, pH 7.5 with 1% (*v*/*v*) Triton X-100, 2% (*w*/*v*) sodium deoxycholate and 2% (*w*/*v*) SDS; all Sigma-Aldrich, St. Louis, MO, USA) and immediately before use, phenylmethylsulfonyl fluoride (PMSF, Sigma-Aldrich, St. Louis, MO, USA) was added to reach final concentration of 1 mM per 1 × 10^6^ cells. The lysates were set to concentration of 5 × 10^4^ cells in 20 µL. Samples were analyzed by the ELISA as described before [21]. The results obtained in the experiments are expressed as nmol of HNE-His/mg of proteins.

### 2.10. Analysis of Nuclear Morphology with 4′,6-diamidino-2-phenylindole

Cells were prepared the same way as for MTT assay and plated on an 8-well Nunc chamber slide (Sigma, St. Louis, MO, USA). Differentiated and undifferentiated HOS were treated with different concentrations of HNE (0, 1, 5, or 10 μM) as described above. After 24 h cells were fixed with 4% formaldehyde. Cells were then incubated with 0.3 µM 4′,6-diamidino-2-phenylindole (DAPI) solution in PBS for 5 min, rinsed with distilled water, and air-dried in the dark. Slides were mounted with glycerol and were scored blind for gross nuclear morphology under fluorescence microscope (Zeiss Axiovert25, HBO 50 Oberkochen, Germany). Morphology evaluation included scoring for nucleus size, chromatin condensation (condensation of chromosomes and necrotic shrinkage of chromatin), and presence of micronuclei, providing information about cell cycle phases, apoptosis, and necrosis, as described before [15,22].

### 2.11. ROS Measurement

Cellular ROS production was measured by a method based on oxidation of 2,7-dichlorodihydrofluorescein diacetate (DCFH-DA, Fluka, Charlotte, NC, USA) to the fluorescent compound 2,7-dichlorofluorescein (DCF). This probe is highly reactive with hydrogen peroxide and has been used to evaluate ROS generation in cells [23,24]. HOS cells were seeded in white 96-well plates at density of 2 × 10^4^ cells per well in DMEM supplemented with 5% FCS 2 h prior to treatment. After 2 h, medium was removed, the cells were washed with Hanks balanced salt solution (HBSS) and incubated with 10μM DCFH-DA in the HBSS. After DCFH-DA was removed, the cells were washed and incubated with HBSS buffer with different concentrations of HNE (0, 1, 2.5, 5, or 10 μM HNE) for 2 h and the fluorescence intensity was measured with a Varian fluorescence spectrophotometer (Varian, Palo Alto, CA, USA) with an excitation wavelength of 500 nm and emission detection at 529 nm and under fluorescence microscope (Zeiss Axiovert25, HBO 50, Oberkochen, Germany). Results are expressed as relative fluorescence units (RFU). 

### 2.12. Immunocytochemical Fluorescence Labeling for c-FOS and c-MYC

Cells were prepared the same way as for MTT assay and plated on an 8-well Nunc chamber slide (Sigma, St. Louis, MO, USA) and left to attach for 6 h. Differentiated and undifferentiated HOS were treated with different concentrations of HNE (0, 1 μM) as described above. After 24 h cells were fixed 2 min with methanol and stored in 4% buffered formaldehyde until analysis. Samples were washed 3 × 5 min with PBS before immunocytochemistry. 

C-MYC immunostaining was performed with primary antibody against c-MYC (SC-42 Santa Cruz Biotechnology, Dallas, TX, USA) diluted 1:50 with 1% of bovine serum albumin (BSA) in PBS incubated overnight at ±4 °C. After that, samples were washed with 3 × 5 min with PBS and overlaid with secondary antibody labelled with Texas red (TR, sc 3797 Santa Cruz Biotechnology, Dallas, TX, USA) diluted 1:100 in 1% BSA with PBS for 2 h. Samples were washed 3 × 5 min with PBS, mounted in glycerol, and analyzed under fluorescence microscope (Zeiss Axiovert25, HBO 50, Oberkochen, Germany) with ImageJ software (NIH and LOCI, Bethesda, WI, USA). Results are expressed as fluorescence intensity.

C-FOS immunostaining was performed with primary antibody against c-FOS (F7799 Sigma-Aldrich, St. Louis, MO, USA) diluted 1:100 with 1% of bovine serum albumin (BSA) in PBS incubated overnight at ±4 °C. After that, samples were washed with 3 × 5 min with PBS and overlaid with secondary antibody labeled with fluorescein isothiocyanate (FITC, F1262 Sigma, St. Louis, MO, USA) diluted 1:100 in 1% BSA with PBS for 2 h. Samples were washed 3 × 5 min with PBS, mounted in glycerol and analyzed under fluorescence microscope (Zeiss Axiovert25, HBO 50, Oberkochen, Germany) with ImageJ software (NIH and LOCI, Bethesda, WI, USA). Results are expressed as fluorescence intensity.

### 2.13. Proteasomal Activity

Cells were grown in T25 cell culture flasks (Sarstedt, Nümbrecht, Germany), trypsinized, centrifuged 5 min at 200× *g*, and supernatant was discarded. Cell pellets were resuspended in 200 µL cold (4 °C) lysis buffer (250 mM saccharose, 25 mM HEPES, 10 mM MgCl_2_, 1 mM EDTA, pH 7.8 with 1 mM dithiothreitol, DTT, Carl Roth, Karlsruhe, Germany) and lysed on shaker at highest speed for 1 h at 4 °C. Samples were then centrifuged for 30 min at 3000× *g* at 4 °C. Supernatants were used for determination of 26S proteasomal activity. Reaction mix consisted of 10 µL of sample; 33.3 µL of incubation buffer (450 mM TRIS, 90 mM KCl, 15 mM Mg-acetate, 15 mM Mg-chloride, pH 8.2); 0.2 µL of 0.5 M DTT; 61.5 µL of H_2_O, 4.5 µL 531 µM Lactacystin (Sigma-Aldrich, St. Louis, MO, USA). Reaction mix was left for 10 min, and then 5 µL of 100 mM ATP and 10 µL of 2 mM Suc-LLVY-MCA (Bachem, Bubendorf, Switzerland) fluorogenic peptide substrate was added. Standards were prepared by dissolving 7-Amino-4-methylcoumarin (MCA, Sigma-Aldrich, St. Louis, MO, USA). Reaction was then incubated in dark at 37 °C 30–60 min. Results were analyzed by plate reader fluorometer with λ_ex_ 360 nm and λ_em_ 460 nm [25].

### 2.14. Fatty Acid Analysis

For the analysis of fatty acid composition, undifferentiated and differentiated HOS were grown in flasks and triplicates of two different cultures were prepared. Cells were trypsinized, washed, counted, and 1 × 10^7^ cell was used for fatty acid analyses. Lipids were extracted in chloroform:methanol (2:1) according to Folch [26]. Heptadecanoic acid C17:0 was added as an internal standard (IS). Fatty acid methyl esters (FAME) were prepared by transesterification with 14% (*v*/*v*) boron trifluoride and dissolved in petroleum ether. Fatty acid methyl esters were analyzed with GC-MS, using a Trace GC and a DSQ mass spectrometer (Thermo, San Jose, CA, USA). Separation was performed on a DB-5MS column (60 m, ID 0.32 mm, 0.25 µm film thickness) (Agilent, Waldbronn, Germany), helium was used as carrier gas and a temperature gradient from 130 to 250 °C within 50 min was applied. Data analysis was done with Xcalibur 1.4 software (Thermo, San Jose, CA, USA) and the NIST library for spectrum identification [27,28]. 

### 2.15. Statistical Analysis

All assays were carried out in triplicates unless otherwise stated for each particular method. The comparison of the mean values was done using Student’s *t*-test considering values of *p* < 0.05 as significantly different. 

## 3. Results

### 3.1. Characterization of Undifferentiated and Differentiated HOS

**Figure 1:**
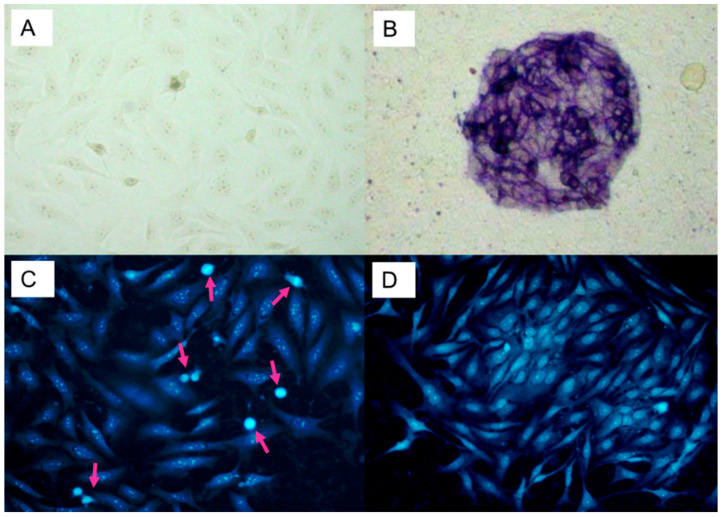
Differentiated and undifferentiated HOS: alkaline phosphatase staining of (**A**) undifferentiated HOS and (**B**) differentiated HOS (blue staining of nodules indicate alkaline phosphatase positive cells); DAPI staining of (**C**) undifferentiated HOS and (**D**) differentiated HOS (arrows point to the mitotic cells).

Differentiated and undifferentiated HOS cells stained for the presence of alkaline phosphatase (ALP) are presented in Figure 1A,B. Undifferentiated HOS cell cultures (Figure 1A) were not stained blue indicating lack of ALP activity. Differentiated HOS cell cultures (Figure 1B) showed blue nodules, which indicated presence of ALP activity. 

DAPI staining of differentiated and undifferentiated HOS are presented in Figure 1C,D. Undifferentiated HOS cell cultures (Figure 1C) had high numbers of mitotic cells and presence of these cells is indicated by pink arrows, while differentiated HOS cell cultures had low numbers of mitotic cells. Differentiated and undifferentiated HOS cells are different (Table 1) with respect to the number of alkaline phosphatase positive nodules in cell culture (*p* < 0.0002), mitosis (*p* < 0.001), and ^3^H-thymidine incorporation (*p* < 0.05). Apoptotic cells were not detected in either of the cell cultures.

### 3.2. GSH, ox GSH, GST, and Proteasomal Activity in Undifferentiated and Differentiated HOS

**Figure 2:**
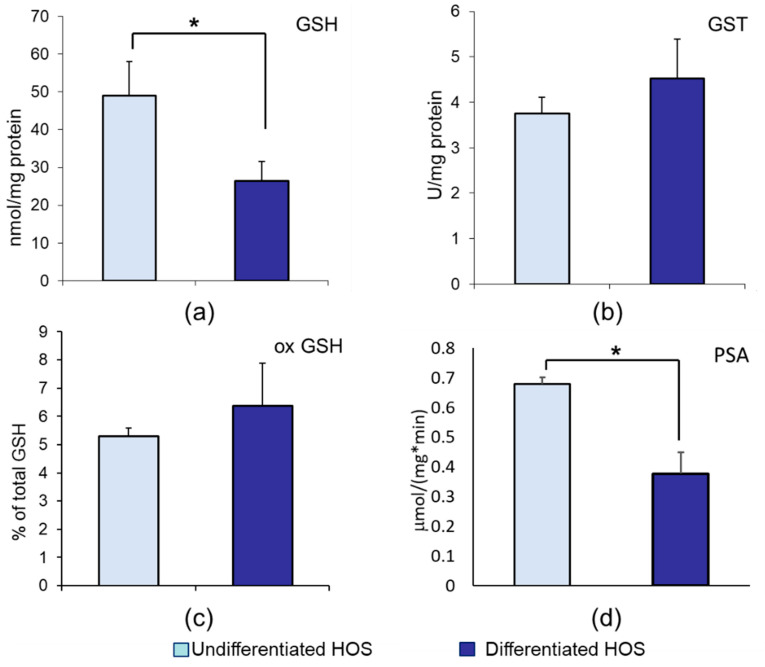
Antioxidative parameters in differentiated and undifferentiated HOS, indicating ability to reduce HNE damage: (**a**) GSH; (**b**) GST activity; (**c**) ox GSH; (**d**) proteasomal activity (PSA). Difference according to *t*-test significant between two cultures, * *p* < 0.05.

Total glutathione (GSH) content in cell cultures expressed in nmol/mg of cellular proteins is presented in Figure 2A. Differentiated HOS contained lower amount of GSH than undifferentiated HOS (*p* < 0.05). Content of oxidized GSH in cell cultures is presented in Figure 2C. Both cell cultures had level of oxidized glutathione below 10% of total GSH and it was not different between undifferentiated and differentiated HOS (*p* > 0.05). Glutathione transferase (GST) activity expressed in U/mg of cellular proteins is presented in Figure 2B. Undifferentiated and differentiated HOS cell cultures had the same GST activity (*p* > 0.05). Proteasomal activity was calculated as activity in µmol/(mg*min) and presented in Figure 2D. Differentiated HOS had lower proteasomal activity than undifferentiated HOS (*p* < 0.05).

### 3.3. Cell Viability, Proliferation, Mitosis, Apoptosis, and Necrosis after Treatment with HNE

**Figure 3:**
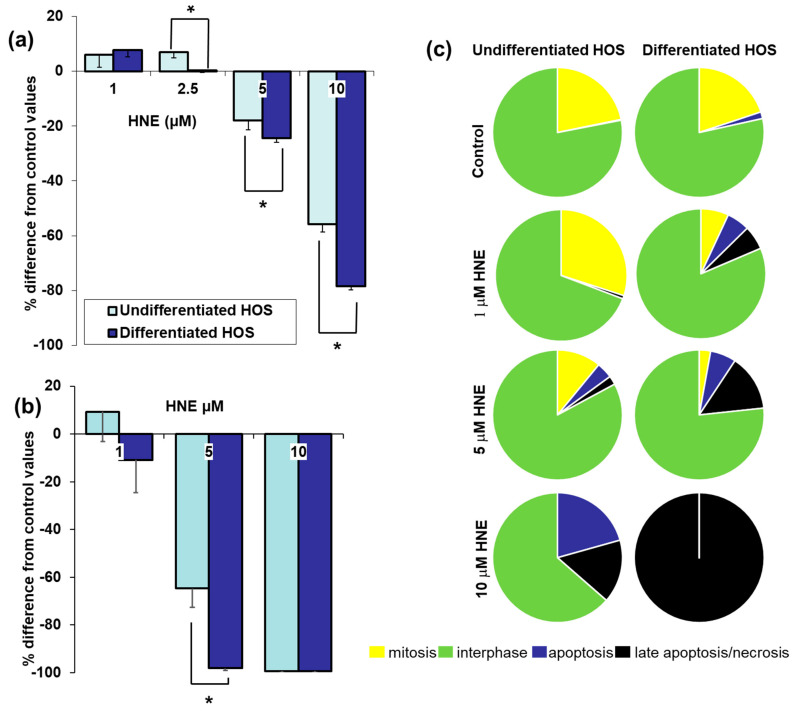
Viability, proliferation, and cell cycle events in differentiated and undifferentiated HOS: (**a**) cell viability (MTT) after HNE treatment; (**b**) proliferation (^3^H-T) after HNE treatment; (**c**) phases of cell cycle in cells treated with HNE. Difference according to *t*-test significant between two cultures, * *p* < 0.05.

Cell viability of HOS treated with different concentrations of HNE is presented in Figure 3A. Low concentrations of HNE (1 µM, 2.5 µM) did not show differences when compared to control, untreated cells (*p* > 0.05), while higher concentrations of HNE (5 µM, 10 µM) significantly decreased viability in both, undifferentiated and differentiated HOS cells (*p* < 0.05). While there was no difference between viability of differentiated and undifferentiated HOS treated with 1 µM HNE, differentiated HOS had significantly lower viability when treated with 2.5 µM, 5 µM, and 10 µM HNE (*p* < 0.05). 

Proliferation of HOS treated with different concentrations of HNE is presented in Figure 3B. Similarly to cell viability assay, both undifferentiated and differentiated HOS treated with 1 µM HNE did not show any differences when compared to control, untreated cells nor compared to each other (*p* > 0.05). Higher concentrations of HNE (5 µM, 10 µM) significantly decreased proliferation in both, undifferentiated and differentiated HOS compared to the control (*p* < 0.05). Treatment with 5 µM HNE significantly decreased proliferation of differentiated HOS compared to undifferentiated HOS (*p* < 0.05). Treatment with 10 µM HNE completely blocked proliferation of both undifferentiated and differentiated HOS (*p* > 0.05).

Effects of different concentrations of HNE on distribution of different phases of cell cycle are presented in Figure 3B. In control cultures, undifferentiated HOS cells had high number of mitosis (22 ± 4%). Control cultures of differentiated HOS cells recovered mitotic activity when trypsinized and plated at low density (20 ± 6%). While undifferentiated HOS were slightly stimulated by 1 µM HNE, differentiated HOS significantly decreased mitotic index (6 ± 3%, *p* < 0.05), had similar reaction pattern as treatment with 5 µM HNE (mitotic index 3 ± 4%, apoptosis 6 ± 6%, necrosis 14 ± 10%). Unlike differentiated HOS, undifferentiated HOS treated with 5 µM HNE had higher mitotic index (11 ± 3%) and lower percentage of necrotic cells (2 ± 1%). Finally, while treatment with 10 µM HNE caused necrosis of differentiated HOS, undifferentiated HOS had 21 ± 5% apoptosis and 16 ± 6% necrosis/late apoptosis. 

### 3.4. HNE Metabolism, GSH Content, Formation of HNE-Protein Adducts

**Figure 4:**
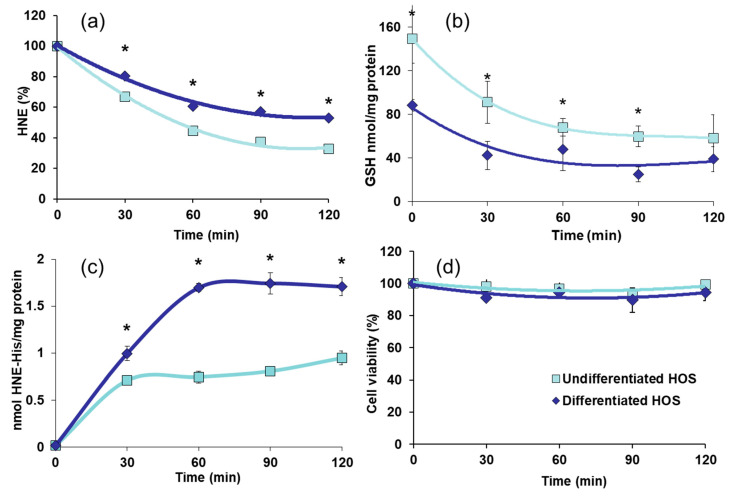
Treatment of undifferentiated and differentiated HOS with HNE (20 nmol/10^6^ cells). (**a**) elimination of HNE from the media; (**b**) content of GSH in cells treated with HNE; (**c**) amount of HNE-protein adducts in cell cultures treated with HNE; (**d**) cell viability checked through the time of experiment upon trypan blue exclusion assay. Difference according to *t*-test significant between two cultures, * *p* < 0.05.

Effects of concentration of HNE 20 nmol/10^6^ cells on HNE metabolism, GSH content, formation of HNE-protein adducts, and cell viability in undifferentiated and differentiated HOS cell cultures are presented in Figure 4.

Free HNE in cell cultures supernatants is presented in Figure 4A. Undifferentiated and differentiated HOS decreased initial HNE concentration already after 30 min (*p* < 0.05 for both cultures). Undifferentiated HOS were more efficient in decreasing free HNE to 40% of initial value after 120 min (*p* < 0.05) while in the same period differentiated HOS decreased HNE to 60% of initial value (*p* < 0.05). Kinetic of GSH in undifferentiated and differentiated cell cultures after treatment with HNE are presented in Figure 4B. GSH decreased during observed period of 120 min in both undifferentiated and differentiated HOS (*p* < 0.05). Concentration of GSH remained higher in undifferentiated cells throughout the observed period of 90 min (*p* < 0.05). HNE-protein adducts formed after treatment with HNE are presented on Figure 4C. In undifferentiated HOS cells HNE-protein adducts increased until 30 min when they reached plateau, while in differentiated HOS the plateau was reached after 60 min. Amount of HNE-protein adducts in differentiated HOS cell cultures was higher than in undifferentiated (*p* < 0.05). Cell viability evaluated upon trypan blue exclusion assay presented in Figure 4D shows that both undifferentiated HOS and differentiated HOS were viable throughout the whole experiment, (*p* > 0.05).

### 3.5. ROS Production in Cells after Treatment with HNE

**Figure 5:**
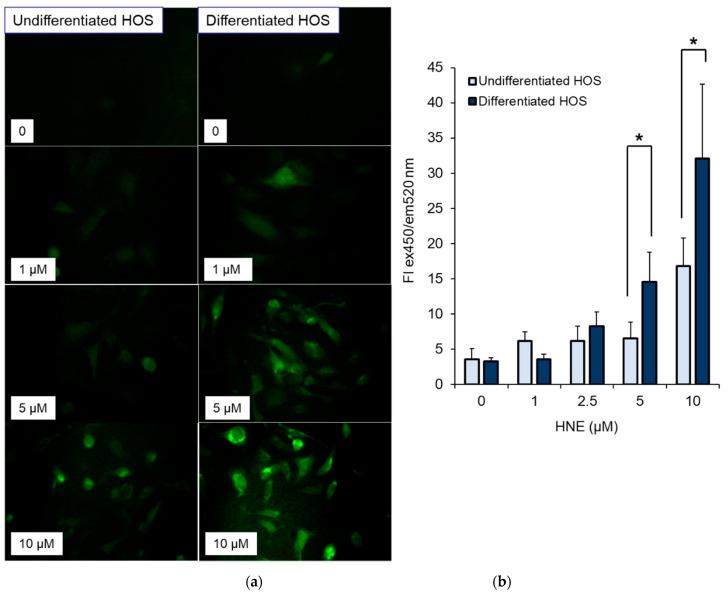
Induction of ROS in cell cultures treated with HNE. (**a**) Green fluorescence as a measure of ROS in differentiated and undifferentiated HOS treated with HNE; (**b**) fluorescence intensity in undifferentiated and differentiated HOS analyzed by *t*-test, * *p* < 0.05.

ROS production in undifferentiated and differentiated HOS 2 h after treatment with different concentrations of HNE (0, 1, 2.5, 5, and 10 µM) is presented in Figure 5. Green fluorescence indicated presence of ROS in cell cultures. HNE caused concentration-dependent increase in ROS production in both cell cultures (*p* < 0.05 for 5 and 10 µM HNE). More ROS was present in differentiated HOS cell cultures (*p* < 0.05 for 5 and 10 µM HNE). 

### 3.6. c-FOS and c-MYC in Undifferentiated and Differentiated HOS

**Figure 6:**
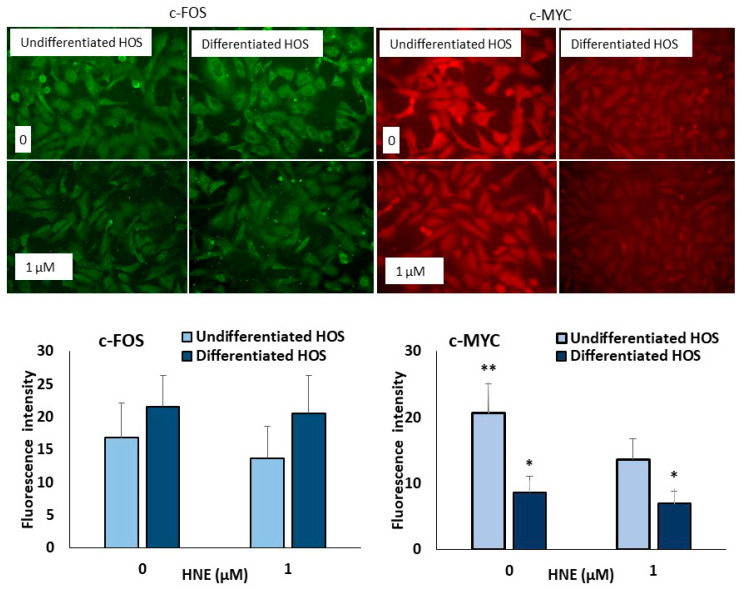
Representative immunofluorescence labeling for c-FOS and c-MYC in differentiated and undifferentiated HOS. Difference according to *t*-test significant between two cultures, * *p* < 0.05; and 0 and 1 µM HNE, ** *p* < 0.05.

Immunocytochemical fluorescent staining for c-FOS and c-MYC is presented in Figure 6. There is no difference in the intensity of c-FOS staining in control cultures for differentiated and undifferentiated HOS (*p* > 0.05). The intensity of c-FOS positivity is the same in both cultures, differentiated and undifferentiated HOS when treated with 1 µM HNE (*p* > 0.05). Unlike c-FOS, undifferentiated HOS have higher c-MYC positivity in both control and HNE treated cultures compared to differentiated HOS (*p* < 0.05). C-MYC positivity decreased in undifferentiated HOS when treated with 1 µM HNE (*p* < 0.05). 

### 3.7. Fatty Acids Composition in Undifferentiated and Differentiated HOS 

**Figure 7:**
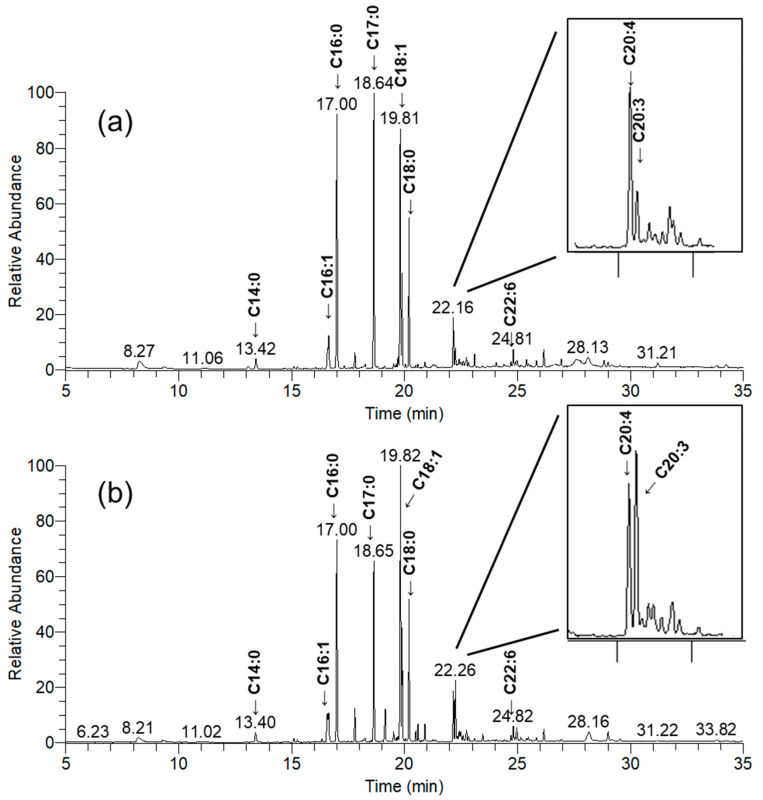
Representative GC chromatograms of fatty acids extracted from (**a**) undifferentiated HOS and (**b**) differentiated HOS cell culture. Fatty acids detected in HOS are labeled on chromatograms. Differences in chromatographs are extracted for each sample.

Samples of fatty acids chromatograms of undifferentiated and differentiated HOS cell cultures separated by GC are presented in Figure 7. Differentiated cell cultures have one peak higher and this was designated on a chromatogram. Based on the obtained data, peaks eluting at the selected retention times were further analyzed by the MS. Fatty acids determined by GC-MS in cell culture samples are presented in Table 2. The most abundant fatty acids were palmitic acid C16:0, oleic acid C18:1, and stearic acid C18:0, although they did not significantly differ between two cultures (*p* > 0.05 for all). We observed very small amounts of C18:2 in both undifferentiated and differentiated HOS as three peaks (RT 19.52, 19.64, 19.71 min), but exact structures we could not determine so those data are not presented in the table. 

**Figure 8:**
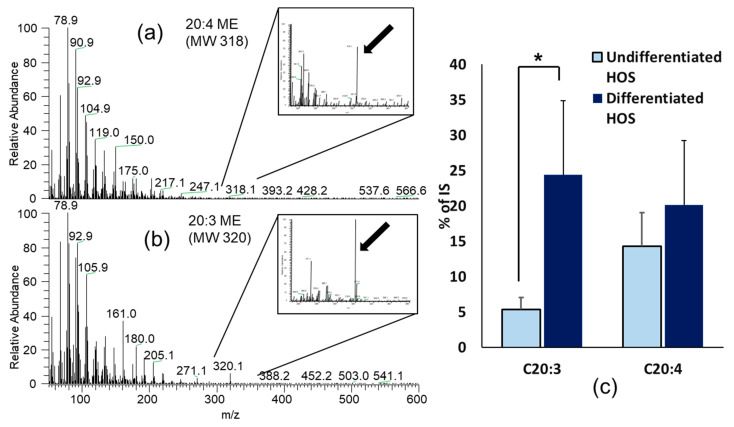
Fatty acid analyses. (**a**) Arachidonic acid, 20:4 (MW 318) and (**b**) mead acid 20:3, (MW 320); (**c**) amounts of 20:3 and 20:4 in undifferentiated and differentiated HOS normalized with internal standard. Amount of C20:3 was different between cultures, * *p* < 0.05.

In agreement with the initial screening performed by GC-FID one fatty acid was found to significantly differ between differentiated and undifferentiated HOS cells (Figure 8). Differentiated HOS cells have significantly increased level of 5,8,11-eicosatrienoic acid C20:3 n-9 (*p* < 0.05). The amounts of other fatty acids were not different between cultures (*p* > 0.05).

## 4. Discussion

Evidence suggest that normal cells are less sensitive to HNE than corresponding malignant cells, such as normal human peripheral lymphocytes compared to CEM-NKR leukemic cells [13] and normal mesenchymal cells such as human osteoblasts and WI38 fibroblasts compared to malignant 143B and HOS osteosarcoma cells [14]. Therefore, we wanted to investigate how the process of differentiation affects sensitivity of malignant cells to HNE. For this purpose, we used HOS osteosarcoma cells as they are able to differentiate in cell culture. When HOS cells reach confluence, they start to differentiate and express alkaline phosphatase as functional and morphological biomarker of osteogenic differentiation [15]. These cells retained viability and do not undergo apoptosis, but were able to proliferate when seeded at lower density and even completely recover ability to proliferate reaching the same percentage of mitosis in the culture as undifferentiated HOS. If the cultures are regularly maintained, it is not expected that deprivation of nutrients is caused. Similar effect is observed on adipocytes where contact inhibition and growth arrest cause differentiation [29]. It is important to notice that HNE itself is also a growth regulating factor which can induce differentiation in different cell types [30,31]. In HOS model we used HNE decreased differentiation when added repeatedly every second day for 10 days [15]. 

Therefore, we expected that differentiated HOS cells will exert characteristics of osteoblasts because of the presence of ALP, and will be more resistant to HNE due to our previous results on mesenchymal cells [14]. 

Firstly, we performed MTT assay and ^3^H-thymidine proliferation assay which, surprisingly, showed that differentiated HOS are more sensitive to HNE. They also had more apoptotic cells and late apoptotic/necrotic cells than undifferentiated HOS. Furthermore, we made immunostaining HOS cells for the two transcription factors, c-FOS and c-MYC. The results of c-FOS presence in the cells are in the agreement on HL-60 cells where HNE does not affect c-FOS [32], but inhibits c-MYC [33] indicating that this decrease can be the cause of lower proliferation and viability at higher HNE concentrations. Interestingly, c-MYC showed to be differently present in differentiated and undifferentiated HOS. Overexpression of c-MYC increases proliferation of mesenchymal stem cells [34]. As c-MYC is a transcription factor which activates genes involved in proliferation [35], this result supports lower proliferation index in differentiated HOS. 

One of the possible explanations of those results is that differentiated HOS probably reached senescence. In support of this possibility is the data showing that senescent chondrocytes are more sensitive to oxidative stress [36]. We expected that one of the factors causing different sensitivity to HNE could be the changes in reduced glutathione (GSH) level. GSH is an important intracellular protector against free radicals as well as radical scavenger responsible for HNE detoxification [37]. 

The GSH content in differentiated and undifferentiated HOS was measured showing that differentiated HOS cells had lower levels of GSH, which might explain their higher sensitivity to HNE. Levels of oxidized GSH were below 10% in both nondifferentiated and differentiated HOS cell cultures, showing that those cells were equally viable. The process of HOS cells differentiation was associated with changes in cell metabolism resulting in decreased GSH content in those cells. GSH content in cells is different depending on the cell cycle: it increases from G phase through S phase and reaches maximum at G2/M phase [38]. Differentiated osteosarcoma cells had lower number of mitotic cells, thereby agreeing with this study. Some malignant cells like hepatoma have higher GSH than normal hepatic cells [39], while nonmalignant mesenchymal cells have higher amounts of GSH than osteosarcoma cells [14]. Differences in GSH content appear to play an important role in cellular sensitivity to HNE. 

Treatment with HNE additionally decreased GSH content in cells [37]. Lower content of GSH and its consumption resulted in higher HNE-protein adducts in differentiated HOS cells. Previously, we presented linear correlation between GSH content and HNE-protein adducts formed in cells after exposure to HNE [14]. After a certain period of time, both GSH level and HNE-protein adducts reached equilibrium, regardless of the free HNE in cell supernatant and free GSH. In this equilibrium state GSH and HNE-protein adducts reached plateau. One of the possibilities for these results is the method for measuring HNE-protein adducts, which detects only HNE-histidine adducts, but not other modifications, such as lysine, cysteine, or arginine [40]. HNE-protein adducts are formed in vivo in cancer cells and normal tissue and change during tumor progression. Depending on tumor origin and stage, formation of adducts could be lower, the same, and higher than corresponding normal tissue [41,42]. 

HNE is a substrate of glutathione S-transferases (GST) [43], a family of enzymes that catalyzes the conjugation of chemicals to glutathione. Two isozymes of α-class of GSTs, hGSTA4-4 and hGST5.8 have high catalytic affinity for HNE [44,45]. GS-HNE conjugates are exported from the cells by active ATP-dependent transport through RLIP76 protein [46]. We measured total glutathione transferase activity in HOS and there was no difference between differentiated and undifferentiated HOS. Overexpression of GSTs is related to an increase in resistance to anticancer drugs or alkylating agents [47,48]. In keratinocytes, HNE metabolites are determined by MS; 48% are attributed to two unconjugated metabolites created by aldehyde dehydrogenase [49] and 52% are four metabolites created by conjugation with GSH further metabolized by oxido-reductive enzymatic processes. In erythrocytes, 70% of metabolites are conjugates with glutathione and 25% one of the unconjugated metabolites [50]. It seems that cells of different origin differ in preference of metabolic pathways which is used for HNE degradation. Earlier, we determined linear correlation between HNE-modified proteins and GSH content in mesenchymal cells [14], so we supposed that these cells use GSH preferentially to eliminate HNE. 

Proteasomes are responsible for degradation of damaged proteins and its role in tumor progression is not yet clear although there are attempts to use proteasomal inhibitors in tumor treatments [51]. HNE damages proteins which are then substrate for degradation by 20S proteasome subunit of 26S proteasome, responsible also for degrading oxidized proteins. Mildly modified proteins with HNE concentrations such as 1–10 μM are easily degraded by the proteasome, while high concentrations of HNE such as 100 μM extensively modify proteins. This results in formation of protein aggregates which inhibit proteasomal activity [52]. Undifferentiated and differentiated HOS were checked for 26S proteasomal activity and we found that differentiated HOS had lower proteasomal activity which makes processing damaged proteins more problematic. HNE induced production of ROS in undifferentiated and differentiated HOS after treatment in a concentration dependent manner, the higher the HNE concentration used, the higher were the ROS levels measured. In support to GSH results, HNE caused higher increase of ROS in differentiated HOS. The method we used for ROS determination with DCFH-DA is widely used for H_2_O_2_, although there are some controversies about the compound which causes oxidation of this substrate. It is suggested that hydroxy radicals or peroxinitrite could do that also [53]. Fatty acid composition in undifferentiated and differentiated HOS was measured because PUFAs can serve as a substrate for oxidation. Only one fatty acid was found to be significantly different between HOS with different degree of differentiation and this was 5,8,11-eicosatrienoic acid (C20:3 n-9, mead acid) which was found to be higher in differentiated cells.

This particular fatty acid belongs to the group of omega-9 fatty acids and is the only one formed de novo in the body in the state of fatty acid deficiency. Essential fatty acid deficiency (EFAD) is considered when the ratio between triene/tetraene fatty acids is >0.4 [54]. C20:3 is formed from oleic acid when there is restriction in omega-6 fatty acids [55]. The elevated level of C20:3 in differentiated HOS cells could be due to increased expression of enzymes involved in omega-9 fatty acid synthesis. Indeed, studies on NIH3T3 and Hepa1-6 cells demonstrated that Elovl5, Fads1, or Fads2 are involved in synthesis of 20:3 omega-9 fatty acid and their downregulation causes a decrease in C20:3 fatty acid level [56]. Furthermore, EFAD changes composition of fatty acids in bone tissues toward high C20:3 and low C20:4 [57]. Normal, young cartilage has very high content of C20:3 levels and low level of C20:4 [58]. This is supposed to be result of fatty acid deficiency due to low vascularization, and C20:3 is also blocking angiogenesis [59]. However, data are not straightforward because results in growing chicks show very high C20:4 in bone as well as in cartilage, but not C20:3 [60]. Perhaps consummation of food with a higher amount of omega-6 linoleic acid than omega-9 oleic acid causes this [60].

There are no literature data about the role of C20:3 fatty acid in cells of osteogenic origin as well as osteosarcoma cells. It is known that it serves as a substrate for 5-lipooxigenase and is converted into LTA3 which inhibits synthesis of proinflammatory LTB4 [61]. It is supposed to block osteoblasts activity, decreasing ALP activity in osteoblasts [62]. In our study differentiated HOS had both increased alkaline phosphatase activity and more C20:3, therefore, our results do not support this. It is known that addition of other fatty acids such as docosahexaenoic acid n-3 induces apoptosis via ROS production [63]; addition of PUFAs can induce oxidative stress [64] because PUFAs can be autoxidized or enzymatically oxidized by oxidases [65,66]. In our study differentiated HOS had more PUFAs in total, which can increase their sensitivity to cytotoxic activity of HNE. 

The differentiation of HOS was accompanied by a decrease in the ability of HOS cells to metabolize HNE and protect themselves from its toxic effects. Since differentiation of HOS cells was accompanied also by increased production of C20:3 fatty acid, we assume that could make them more subjected to free radical-initiated oxidative chain reactions and more vulnerable to the effects of reactive aldehydes such as HNE. In favor of this possibility are findings of novel, selective anticancer effects of HNE observed for the other cancer cell types [67], related to the lipid metabolism and ROS production by cancer and surrounding nonmalignant cells [68], resembling findings observed also for another reactive aldehyde acrolein [69,70], thus supporting further studies on biomedical relevance of these bioactive markers of lipid peroxidation [71,72].

## Figures and Tables

**Table 1:** Characterization of undifferentiated and differentiated cell cultures.

	Mitosis	Apoptosis	ALP Positive Nodules/10 cm^2^	^3^H-Thymidine
Undifferentiated HOS	23%	0%	0.74 ± 0.64	17,698 ± 1230
Differentiated HOS	3%	0%	37.77 ± 4.84	9814 ± 920
*t*-test	*p* < 0.001		*p* < 0.0002	*p* < 0.05

**Table 2:** Fatty acids detected in cell samples. Values are expressed as a percentage of IS ± sd. Difference according to *t*-test significant between two cultures, * *p* < 0.05.

	Fatty Acid	Undifferentiated HOS	Differentiated HOS
C14:0	Myristic acid (Tetradecanoic acid)	3.03 ± 0.72	3.57 ± 1.27
C16:0	Palmitic acid (Hexadecanoic acid)	74.01 ± 14.45	85.41 ± 32.26
C16:1	Palmitoleic acid (9-hexadecenoic acid)	10.00 ± 3.58	11.56 ± 5.01
C17:0 ^1^	Margaric acid (Heptadecanoic acid)		
C18:1	Oleic acid (9-Octadecenoic acid)	70.88 ± 21.05	108.37 ± 47.25
C18:0	Stearic acid (Octadecanoic acid)	40.72 ± 11.56	59.75 ± 20.63
C20:4	Arachidonic acid (5,8,11,14-Eicosatetraenoic acid)	14.29 ± 4.75	20.11 ± 9.15
C20:3	Mead acid (5,8,11-Eicosatrienoic acid)	5.40 ± 1.65	24.40 ± 10.50 *
C22:6	(4,7,10,13,16,19-Docosahexaenoic acid)	4.33 ± 1.86	6.13 ± 3.07

^1^ Fatty acid added to samples as IS.
